# Poly Aryl Ether Ketones (PAEKs) and carbon-reinforced PAEK powders for laser sintering

**DOI:** 10.1007/s10853-017-0840-0

**Published:** 2017-02-06

**Authors:** Binling Chen, Yuan Wang, Silvia Berretta, Oana Ghita

**Affiliations:** College of Engineering, Mathematics and Physical Science, University of Exeter, Exeter, EX4 4QF UK

**Keywords:** Milling, Carbon Fibre, Composite Powder, Milled Powder, Poly Ether Ether Ketone

## Abstract

This paper discusses various methods of fabrication of plain and carbon-reinforced composite powders, as well as a range of powder characterisation test methods suitable for defining powders for laser sintering. Two milling processes (based on disc blades and rotatory cutting knives) were used as methods of fabrication of powders, starting from injection moulding granule grades, for comparison with current powders obtained directly from polymerisation processes. It was found that the milling process affects the particles properties. The rotary milling produced powders with superior properties in comparison with the disc milling method. Tests including particle size distribution, angle of repose, aspect ratio, sphericity and roundness of particles were employed to compare and assess the suitability of powders for laser sintering. The Brunauer–Emmett–Teller test was identified as a useful method to define surface roughness and porosity of the particles. The carbon fibre (Cf) Poly Ether Ketone (PEK) granules milled well and after an additional sieving process created a good quality powder. This is the first attempt to investigate properties of PEK powder with encapsulated Cf and follow their sintering profile through hot-stage microscopy. It is expected that this type of composite powder will create isotropic structures in comparison with the highly anisotropic properties given by the known dry mix composite powders, currently used in laser sintering.

## Introduction

Laser sintering (LS) is one of the most advanced and promising fabrication methods amongst the polymeric additive manufacturing technologies. This process builds 3D-structured parts by selectively sintering successive layers of powdered material under a laser. Currently, Polyamide (PA) powders dominate the majority of the current market of polymeric materials for LS. New processable polymeric materials could substantially broaden the applications. Recently, a new family of high temperature polymers, Poly Aryl Ether Ketones (PAEKs), has attracted attention for the LS process. Examples are Poly Ether Ketone (PEK) and Poly Ether Ether Ketone (PEEK), with potential applications in many industrial sectors due to their high melting temperature, biocompatibility, excellent wear and chemical resistance [1–3]. Their composites have also been used as metal replacements to further enhance their mechanical or thermal properties [4, 5].

Historically, most studies in laser sintering focused either on the manufacturing process or on the characterisation of the final sintered parts. However, recently, several studies have recognised the importance of powder properties on the sintering process [6–10]. Researchers carried out extensive powder analysis [6–9], or investigated new methods of fabrication of powders and applied milling as an alternative [10].

Ziegelmeier et al. [6, 7] tried to define a relationship between the characteristics of un-sintered powder and the properties of components fabricated by LS. The authors investigated the influence of the bulk and flow behaviour of two types of thermoplastic elastomer polyurethane (TPU) and Duraform Flex (DF) on the resulting properties of the built components. The packing, bulk density and flow efficiency of the powders were examined. They found that better packing and flow efficiency of un-sintered powders can improve the fabrication of components providing enhanced mechanical properties and denser structures.

Berretta et al. [9] firstly attempted to expand the range of engineering polymers for HT-LS. The paper examined the morphology, flowability and particle interactions of two grades of PEEK powders, commercially used for compression moulding and not optimised for the LS process, in parallel with established LS powders such as PA 12 and PEK HP3. The study also analysed the effect of incorporating fillers and additives on the flow behaviour. It was found that the particle morphology had a stronger influence on the flow characteristics and the addition of nanoparticles can improve the powder flow.

Milling is a cost-effective way to create large volumes of powders, of which properties can be tuned to optimise sintered structure [11]. Power properties such as powder particle size, size distribution, particle shape and density can be controlled by milling processing conditions [12–14]. Mys et al. [10] applied rotor milling and ball milling techniques to produce polysulfone (PSU) powders from pellets for selective laser sintering. The powders produced by rotor milling had a desired size and morphology; while the powders produced by ball milling particles exhibited well beyond the desired size range and angular shape. This is the only study reporting milling of high performance polymers for laser sintering. Identifying the optimum method of fabrication of these powders is very challenging as polymers in this category are extremely tough and strong.

As the range of materials for laser sintering is expanding, use of composites is becoming increasingly important for added functionality. Carbon-reinforced nylon powders have been studied by several researchers [15–17]. Goodridge et al. [15] studied laser sintered parts in carbon nanofibres (CNFs)/PA12 powders at 3 wt% CNFs. The authors recognised the importance of a suitable milling process as the cryogenic milled CNFs/PA12 powder did not have a suitable morphology for laser sintering.

Yan et al. [16] prepared the carbon fibre (CF)/PA12 powder through a dissolution-precipitation process at different concentrations: 30, 40 and 50% by weight. Although the powder fabrication method is not further discussed in terms of milling or particle size and shape of the powder, the laser sintered samples of CF/PA12 showed a significant increase in flexural strength and moduli reaching an increase of 114 and 234%, respectively, for the PA12 samples with 50% wt CF. Bai et al. [17] presented an enhancement in PA12 laser sintered parts performance through addition of 0.1% of CNTs using a novel method of coating nylon particles with CNTs. Overall, most studies proved an improvement in properties with addition of various carbon type reinforcement in PA12, the matrix becoming the limiting factor in these materials and their potential applications due to its low glass transition temperature (Tg). Addition of reinforcement in high-temperature polymers such as PEEK comes with new challenges such as increased temperature profiles and risk of degradation and reduced efficiency and quality of milling due to increased toughness and strength of these polymers.

In this paper, we aimed to define the key properties of HT polymeric and composite powders fabricated with two different milling methods for use in HT-LS. As a first step in the laser sintering process, hot-stage microscopy is employed to investigate the rate of sintering of individual particles. The presence of carbon fibres (Cf) and carbon black (CB) within PEEK particles led to a slow neck growth and an initial delay in the coalescence process followed by a rapid increased in the later stages.

## Experimental

### Materials

#### Commercial LS grade powder

The high-temperature LS commercial grade, EOS HP3 PEK, has been purchased from EOS (EOS, Germany) [18].

#### Commercial non-LS grade powder

Victrex PEEK 450PF has been used as the non-LS high-temperature powder. The powder is currently used for compression moulding applications [19].

#### Milled powders

Plain Victrex PEEK 450G granules were milled to a powder using two techniques: disc blade milling and rotary knife milling. The rotary knife milling technique had been used for milling Victrex HT22CA30 PEK granules—a PEK material incorporating 30% Cf; and Victrex 150G903 PEEK granules incorporating Carbon Black (CB) (Victrex, UK) [20].

### Experiments

#### Milling process/sieving

**Figure 1:**
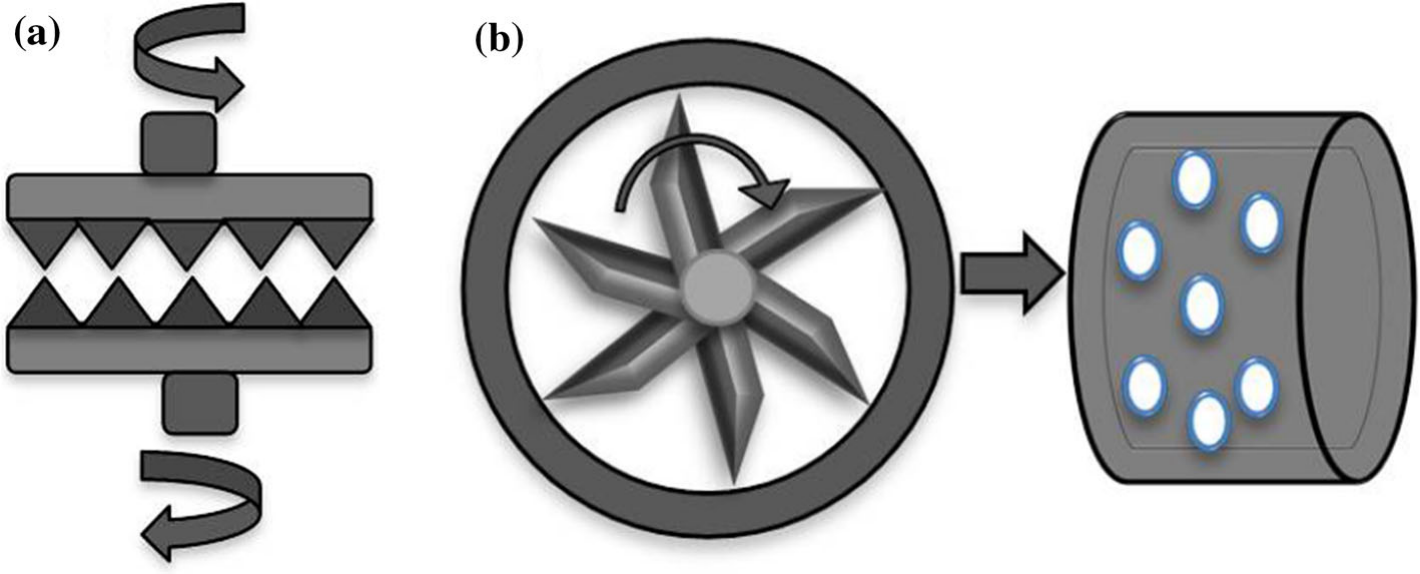
**a** Disc blade milling; **b** rotary knife blade milling

The disc blade milling method used a cryogenic pulverizer (Powder King PKA-18) with a combination of stationary and rotating discs (Fig. 1a). The milling chamber was cooled down with liquid nitrogen to −50 °C before and during milling. In order to achieve the finest powder, the milling gap was set to 0.005′′. The rotation speed was set to 30 Hz. For the rotary knife milling method, a 100UPZII Universal Impact Mill (Hosokawa, Germany) was used to create powders (Fig. 1b). The mill operated at room temperature. The feed material passed from the hopper into the centre of the grinding chamber where it was crushed between the rotation blade and sieves. The sieve mesh size was 2 mm in diameter. The blade rotation speed was 14000 rpm.

The rotary milled powders required additional sieving to eliminate any released small carbon fibres and to reduce the powder particle size to the appropriate distribution. The powders were loaded into a 63 µm mesh and sieved for 1 h.

#### Particle size distribution (PSD)

The particle size distributions of PEK HP3, PEEK 450PF, rotary knife milled PEK/Cf composite and rotary knife milled PEEK/CB were measured by using Malvern Instruments Masterisizer, supported by Microplus Version 2.19 software. The particle size distribution of a sample was measured by detecting its light scattering pattern while the specimen was suspended in 1:1 ethanol/deionized water.

The particle size distributions of disc blade milled and rotary knife milled PEEK 450G could not be measured by using the Malvern Instruments due to their larger particle size. These two materials were measured using a sieve method. A nested column of sieves with wire screen mesh and different mesh sizes (45, 60, 90, 125, 180, 250, 355, 500, 710, 1000 µm) were used for the analysis. Powder was weighted and poured into the top sieve, which had the largest screen mesh size, followed by lower screen sieves placed from top to bottom in a decreasing order. Then, the column was placed in a mechanical shaker and shaken for 60 min. After the shaking was complete, the material on each sieve was weighed. The weight of the sample on each sieve was then divided by the total weight to give a percentage retained on each sieve.

#### Scanning electron microscopy (SEM)

Scanning electron microscopy (SEM) was carried out on a Philips XL-30 machine in a high vacuum mode at an acceleration voltage of 20 kV. Conductive carbon double-sided sticky tape was used to mount the samples on a holder, and the samples were then sputtered with a thin (5 nm) gold coating.

#### Particle shape analysis

The shape analysis was carried out by using the image processing software Image J^®^ [21]. On SEM images in order to numerically evaluate the shape descriptors roundness, circularity, aspect ratio and solidity of the particles under test.

Circularity refers to the shape of a particle. An overall circular polygon has circularity equal to 1, while an elongated shaped element has a value close to zero. Roundness describes the edges and curvatures on the profile of a particle. Particles with round edges have roundness values close to one, while roundness values close to zero indicates the presence of particles with highly sharp edges. Aspect ratio (AR) is the ratio between the major and the minor axes of a particle. Values close to one indicate the presence of equiaxed particles that can correspond to different shapes, i.e. circles, squares, overall circular polygons; while higher values identify highly elongated particles. Solidity is defined as the ratio between the measured area of a particle and its corresponding convex area. Particles such as spheres, rectangles and cubes have solidity values close to one, while particles exhibiting irregularities and protuberances on their surface such as flakes, lumps and outwards elongations have solidity values closer to zero. The equations of the shape descriptors of particle elements are reported in Table 1.

**Table 1:** The equations of the shape descriptors of particle elements

Circularity	Roundness	Aspect ratio (AR)	Solidity
$4\pi \times \frac{\text{Area}}{{\left( {\text{Perimeter}} \right)^{2} }}$	$4 \times \frac{\text{Area}}{{\pi \times \left( {\text{Major axis}} \right)^{2} }}$	$\frac{\text{Major axis}}{\text{Minor axis}}$	$\frac{\text{Area}}{\text{Convex area}}$

The particles were evaluated from the SEM images by using the automatic wand and the freehand tool thus providing the experimental values for the evaluation of the shape parameters in the Image J software. More detail on this analysis is reported elsewhere [22]. For each material, 200–300 particles were analysed.

#### Angle of repose (AOR)

AOR quantifies the angle of a cone of bulk material over a flat surface. The cone is formed by dropping the material through a standard funnel. The angle can be measured between the slant height and the horizontal plane. A small angle of repose indicates high flowability. The test was performed according to the ASTM C144 standard [23]. Each material was tested six times.

#### BET analysis

N_2_ gas sorptions were carried out on a Quantachrome Autosorb-iQ gas sorptometer using conventional volumetric technique. Before gas analysis, the powder sample was evacuated for 3 h at 120 °C under vacuum. The textural properties were determined via N_2_ sorption at −196 °C. The surface area was calculated using the Brunauer–Emmett–Teller (BET) method based on adsorption data in the partial pressure (*P*/*P*o) range of 0.05–0.20.

#### Hot-stage microscopy

Hot-stage microscopy was applied to study particle coalescence under experimental conditions that could simulate the HT-LS process. PEK HP3, PEEK 450PF, rotary knife milled, sieved PEK/Cf composite, and sieved PEEK/CB composite particles were spread on a microscope glass slide and then inserted into the hot-stage device. The powders were heated from room temperature up to 400 °C at 120 °C min^−1^ and held there for 2 min. More details can be found from the literature [24].

According to the literature, PEK HP3 and PEEK 450PF had a coalescence onset temperature of 380 and 340 °C, respectively [24]. Therefore, the starting temperatures were set at 360 °C for PEK and sieved PEK/Cf composite, and at 320 °C for PEEK 450PF and sieved PEEK/CB composite. Four tests were carried out for each material. The ratio of neck length (*x*) to the average particle diameter (*D*) of two particles (*D*1 and *D*2) was measured.

#### Differential scanning calorimetry (DSC)

Thermal properties of powders were also analysed by the Mettler Toledo DSC 821e/700 system. Samples of approximately 8 mg were heated from room temperature to 400 °C at a heating rate of 10 °C × min^−1^ with nitrogen flow of 50 ml × min^−1^. Each sample was repeated three times.

## Results and discussion

### Particle size analysis

**Figure 2:**
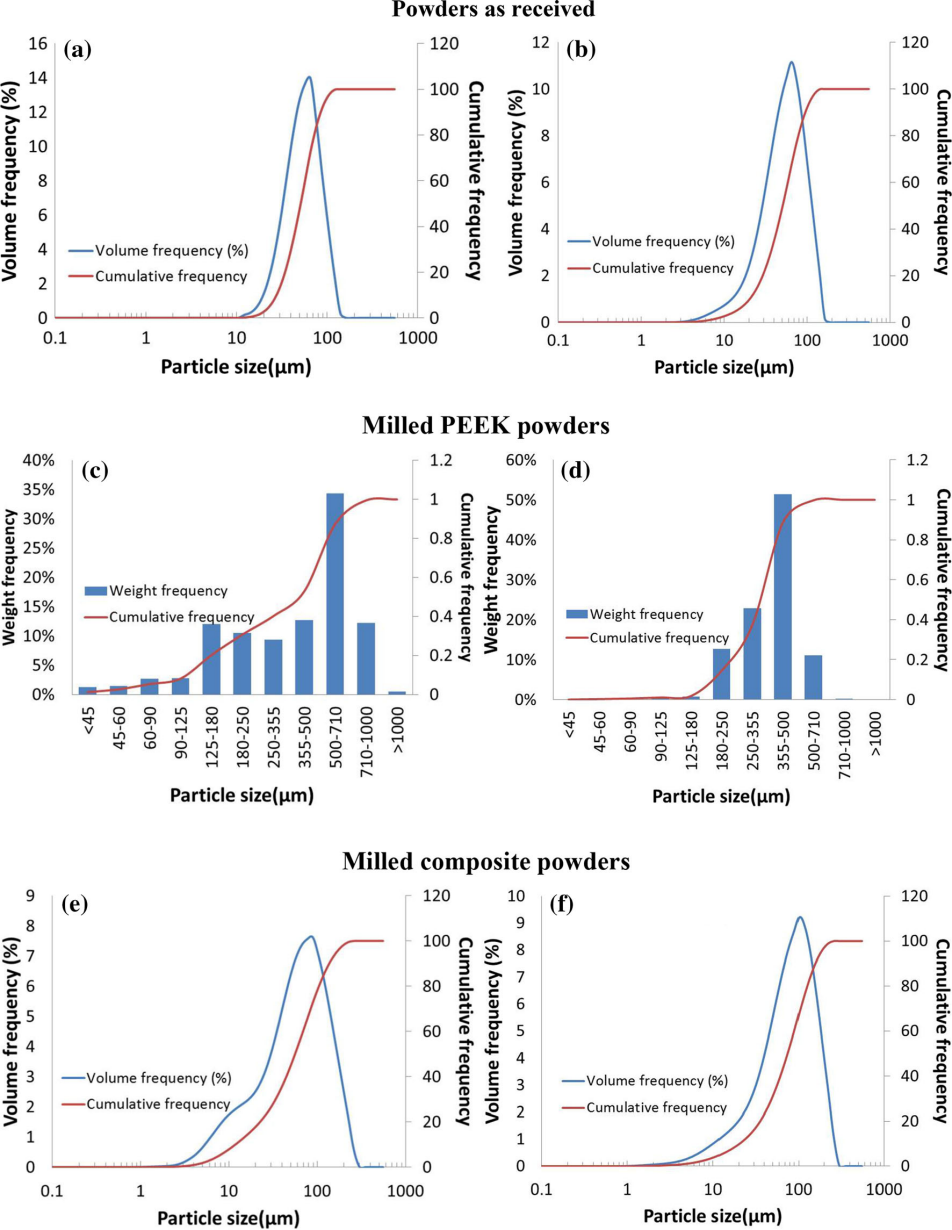
Particle size distribution of **a** PEK HP3, **b** PEEK 450PF, **c** disc blade milled and **d** rotary knife milled PEEK 450G; **e** rotary knife milled PEK/Cf composite and **f** rotary knife milled PEEK/CB

The Particle Size Distributions (PSD) of LS grade powder (PEK HP3), commercial non-LS grade powder (PEEK 450PF), and milled powders (disc blade milled and rotary knife milled PEEK 450G, rotary knife milled PEK/Cf and PEEK/CB composite) are shown in Fig. 2. Both HP3 PEK and PEEK 450PF (shown in Fig. 2a, b) exhibit similar PSDs in a narrow range. However, PEEK 450PF shows a small content of particles below 10 μm. The milled PEEK powders (shown in Fig. 2c, d) show very different distributions with different milling methods. The particles of disc blade milled PEEK 450G are spread in a wide range (45–1000 μm), while the particles of rotary knife milled PEEK 450G have a relatively narrow range (125–710 μm). In addition, rotary knife milled PEK/Cf and PEEK/CB composites (shown in Fig. 2e, f) exhibit similar PSDs in a range of 1–200 μm. The carbon fibre and carbon black particles came off through the milling process. After sieving, the composite particles lower than 63 μm were removed. These PSDs were achieved through one pass of the milling process. If these materials are to be used for LS, the milled powders would need further refining through repeated milling.

### Particle morphology

**Figure 3:**
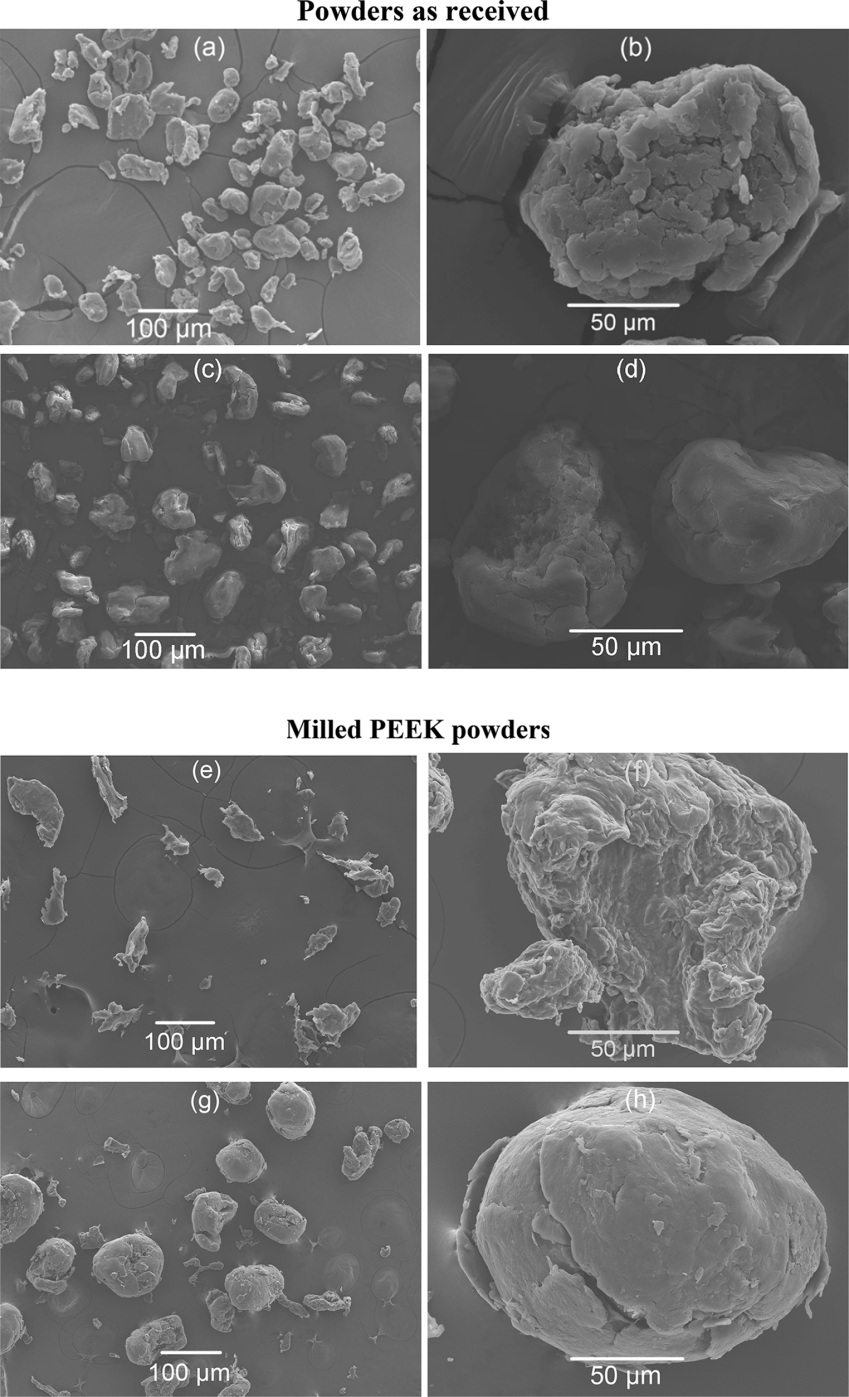
SEM images of PEK HP3 (**a**, **b**); PEEK 450PF (**c**, **d**); disc blade milled PEEK 450G (**e**, **f**); rotary knife milled PEEK 450G (**g**, **h**) under low and high magnifications

**Figure 4:**
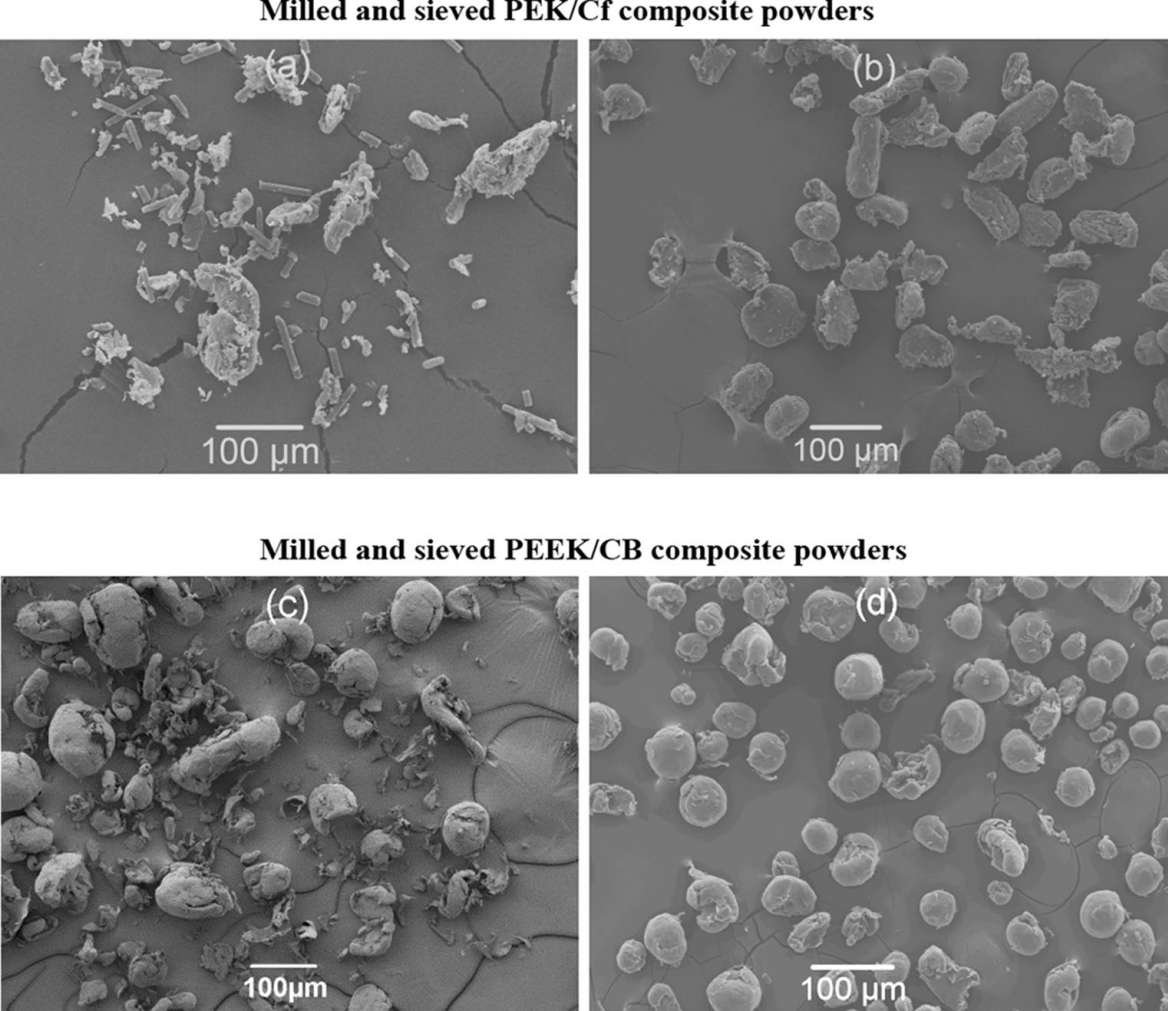
SEM images of rotary knife milled PEK/Cf composite (**a**) and its corresponding sieved composite (**b**); rotary knife milled PEEK/CB composite (**c**) and its corresponding sieved composite (**d**)

The particle morphology of PEEK, PEK powders and their composites are shown in Figs. 3 and 4. Particle morphology is a very important property as previously reported by Berretta et al. [9]. Poor particle morphology can create a rough surface which ultimately leads to poor mechanical performance [22]. Figure 3a–d shows the slightly elongated, round, and sufficiently smooth PEK HP3 and PEEK 450PF particles. The SEM images (Fig. 3e, h) show that disc blade milled PEEK 450G exhibits a less circular shape in comparison with the PEK HP3 powder, where the rotary knife milling method creates round and smooth particles. It is worth noting that the rotary milling process took place at room temperature and most probably the material got hot during the process, which might have led to a softening of the material surface and the creation of the smooth surface finish noticed in the rotary milled particles. In comparison, the disc blade milling which was carried out with liquid nitrogen, created particles with a more angular profile and a rougher surface.

The particle morphology of rotary knife milled composites and their corresponding sieved ones are shown in Fig. 4. After rotary knife milling, large amounts of carbon fibres separated from the polymer particles (shown in Fig. 4a), and those were removed through sieving (shown in Fig. 4b). In contrast, for the same milling technique but for a different material PEEK/CB, the rotary knife milling method produced lots of fibrous PEEK/CB particles as shown in Fig. 4c. Sieving removed the fibrous PEEK/CB particles (Fig. 4d). Therefore, it can be concluded that the particle shapes depend on the types of materials (polymer or polymer composite) as well as the milling process used.

### Particle shape analysis

**Figure 5:**
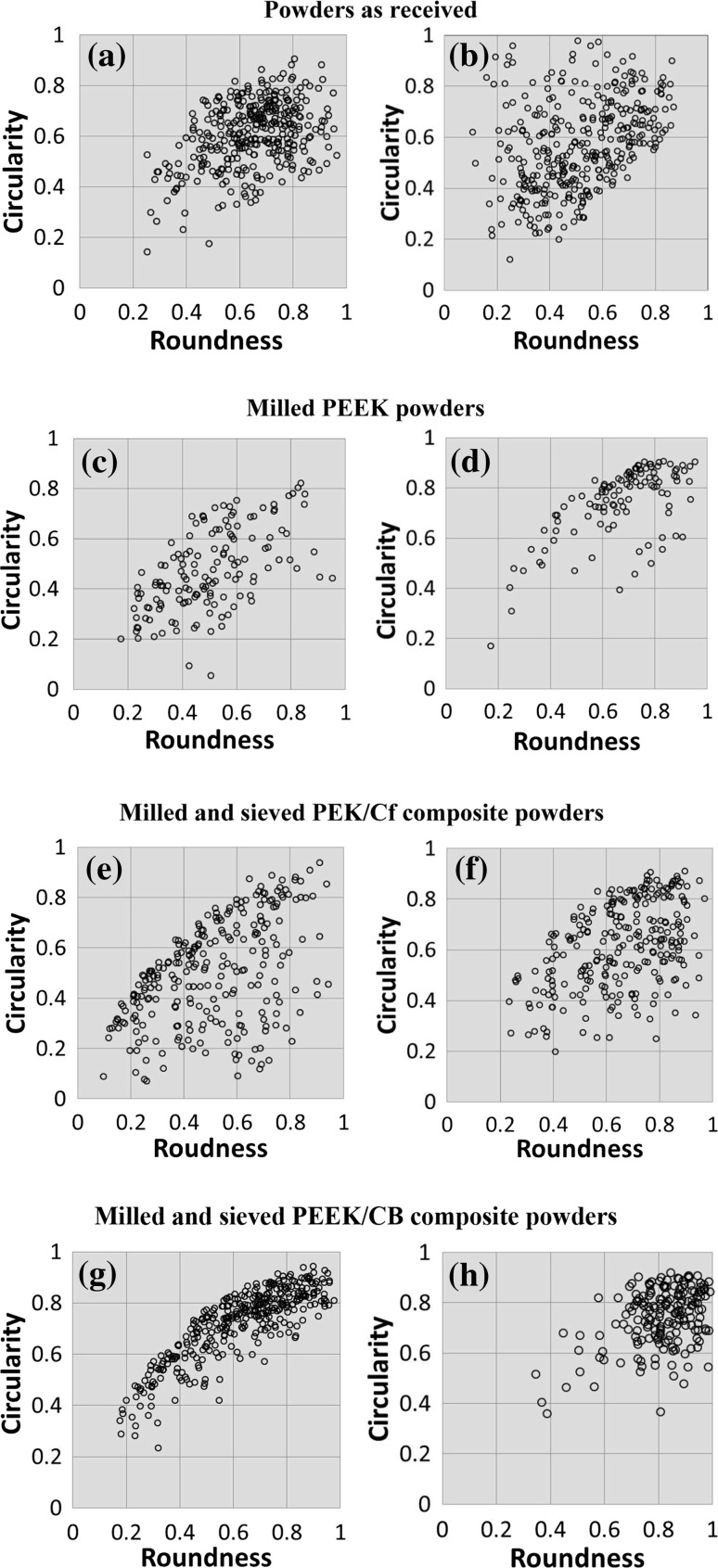
Circularity plotted against roundness of **a** PEK HP3, **b** PEEK 450PF, **c** disc blade milled and **d** rotary knife milled PEEK 450G; **e**, **f** rotary knife milled PEK/Cf composite and **g**, **h** rotary knife milled PEEK/CB (before and after sieving)

**Figure 6:**
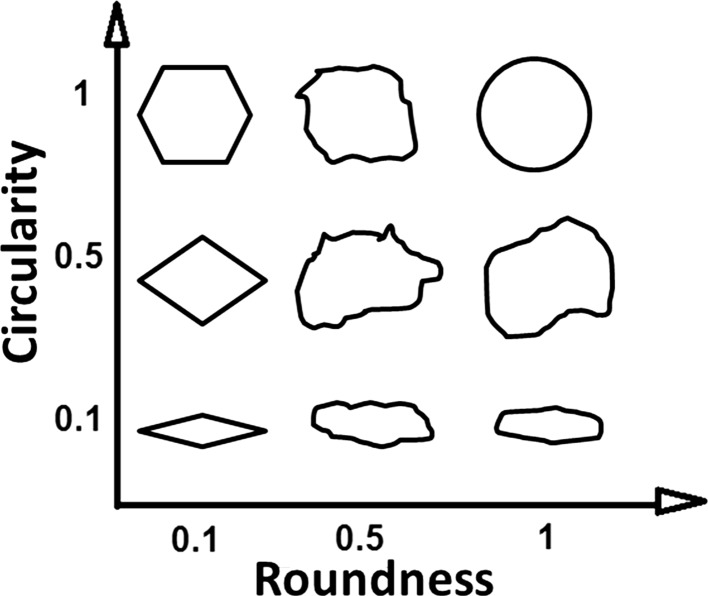
Circularity versus roundness

A combined analysis of shape descriptors circularity and roundness characteristics of the materials was evaluated. A previous study showed that particles with smoother surfaces, rounder and circular shapes can exhibit better flow behaviour [24]. The roundness and circularity datasets of PEEK particles and composites are presented in Fig. 5. The relationship between circularity and roundness is presented in Fig. 6. This graph helps to identify round and spherical particles (high values of circularity and roundness); elliptical particles (low values of circularity but high values of roundness); circular shapes (high value of circularity but low value of roundness) and sharp and elongated shapes (low values of circularity and roundness) [9].

As a commercial material for LS, PEK HP3 exhibits fairly high circularity and high roundness (shown in Fig. 5a), indicating good flowability. Commercial PEEK 450PF shows a wider range of circularity and roundness (shown in Fig. 5b). When using the two milling techniques for the same plain PEEK 450 material, the differences are striking. The disc blade milled PEEK 450 shows a broad range of circularity and roundness (see Fig. 5c), while rotary knife milled shows a relative narrow range and high values of circularity and roundness as it can be seen in Fig. 5d. These results suggest that the rotary knife milled PEEK 450 powder can be successfully used for LS. In the case of PEK/Cf and PEEK/CB composites, adding a sieving step to the standard milling process helps significantly; the circularity and roundness values of the powder are enhanced being comparable with the HP3 PEK values (see Fig. 5e–h). All fibrous particles (Fig. 4c) and loose Cf particles (Fig. 4a) present in the original milled powder are successfully removed through the sieving process. Due to the significant amount of loose Cf, the milled and sieved PEK/Cf powder will have a lower content of Cf than originally present in the granules prior to milling. This will always have to be considered when milling a melt compounded grade of composite.

The previous SEM results combined with the roundness and circularity datasets shown in Fig. 5, confirm again that the particles of PEK HP3, rotary knife milled PEEK 450 and sieved rotary knife milled PEEK/CB composite are the most circular and round powders amongst those analysed.

**Figure 7:**
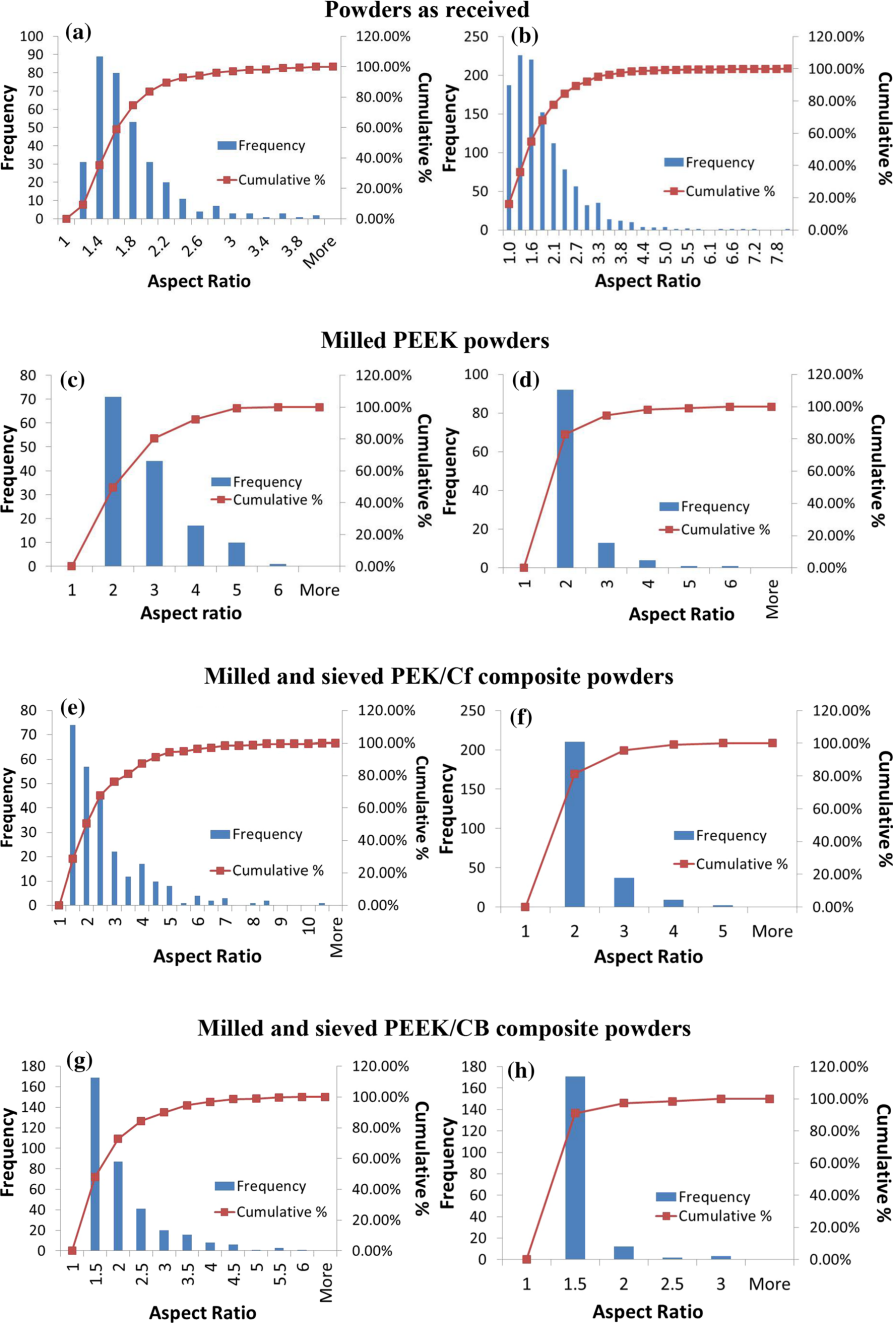
Aspect ratio of **a** PEK HP3, **b** PEEK 450PF, **c** disc blade milled and **d** rotary knife milled PEEK 450G; **e**, **f** rotary knife milled PEK/Cf composite and **g**, **h** rotary knife milled PEEK/CB (before and after sieving)

The Aspect ratio (AR) data as a function of frequency and cumulative percentages are presented in Fig. 7. Aspect ratio is the ratio between the major and the minor axes of a particle as described in experimental methods. It can be seen that HP3 PEK, rotary knife milled PEEK 450 and sieved rotary knife milled PEEK/CB composite exhibit the narrowest distribution amongst all the powders, indicating that the majority of the particles are circular or slightly elongated. As it can be seen, compared with disc blade milled PEEK 450, rotary knife milled PEEK 450 has a narrower distribution. In addition, the unsieved composite powders cover higher AR values up to 10 (Fig. 7e), indicating highly elongated particles compared to the other powders. As expected, the sieving processes narrow the AR by 2–3 in the case of PEK/Cf and 1.5 for PEEK/CB. This is due to the removal of small polymeric particles and broken carbon fibres detached from the bulk of the polymer during milling.

### Flowability results

The results of the AOR tests for PEK HP3, PEEK 450PF, disc blade milled and rotary knife milled PEEK 450G; rotary knife milled PEK/Cf composite and rotary knife milled PEEK/CB (before and after sieving) are listed in Table 2.

**Table 2:** AOR values of PAEK powders and their composite powders

	Material	AOR
Powders as received	PEK HP3	37.8 ± 0.4
	PEEK 450PF	50.4 ± 0.3
Milled PEEK powders	Disc blade milled PEEK 450G	41.9 ± 0.1
	Rotary knife milled PEEK 450G	38.6 ± 0.1
Milled and sieved PEK/Cf composite powders	Rotary knife milled PEK/Cf	52.1 ± 0.1
	Sieved PEK/Cf > 63 µm	39.9 ± 0.1
Milled and sieved PEEK/CB composite powders	Rotary knife milled PEEK/CB	48.1 ± 0.2
	Sieved PEEK/CB > 63 µm	35.9 ± 0.3

As PEK HP3 is an optimised commercial powder for HT-LS, its AOR value is considered an optimal number for HT-LS and it is used as a benchmark material. It can be seen from Table 2 that the milled PEEK 450G shows lower values of AOR than that of the PEEK 450PF (50.4 ± 0.3), the AOR value of disc blade milled and rotary knife milled PEEK 450G is 41.9 ± 0.1 and 38.6 ± 0.1, respectively. It is worth noting that the AOR value of rotary knife milled PEEK 450G is comparable to that of PEK HP3, suggesting the good flowability of rotary knife milled PEEK 450G, although their particle sizes are different. In addition, the values of the sieved composite powders are lower than that of the milled composites. The AOR values of sieved PEK/Cf and sieved PEEK/CB are 39.9 ± 0.1, and 35.9 ± 0.3, respectively. The AOR values of sieved composites are comparable to the optimised commercial powder PEK HP3, although their particle size distributions are different. This is an extremely encouraging result, as PEK/Cf composites are of great interests for strong and light-weight applications.

### BET analysis

**Figure 8:**
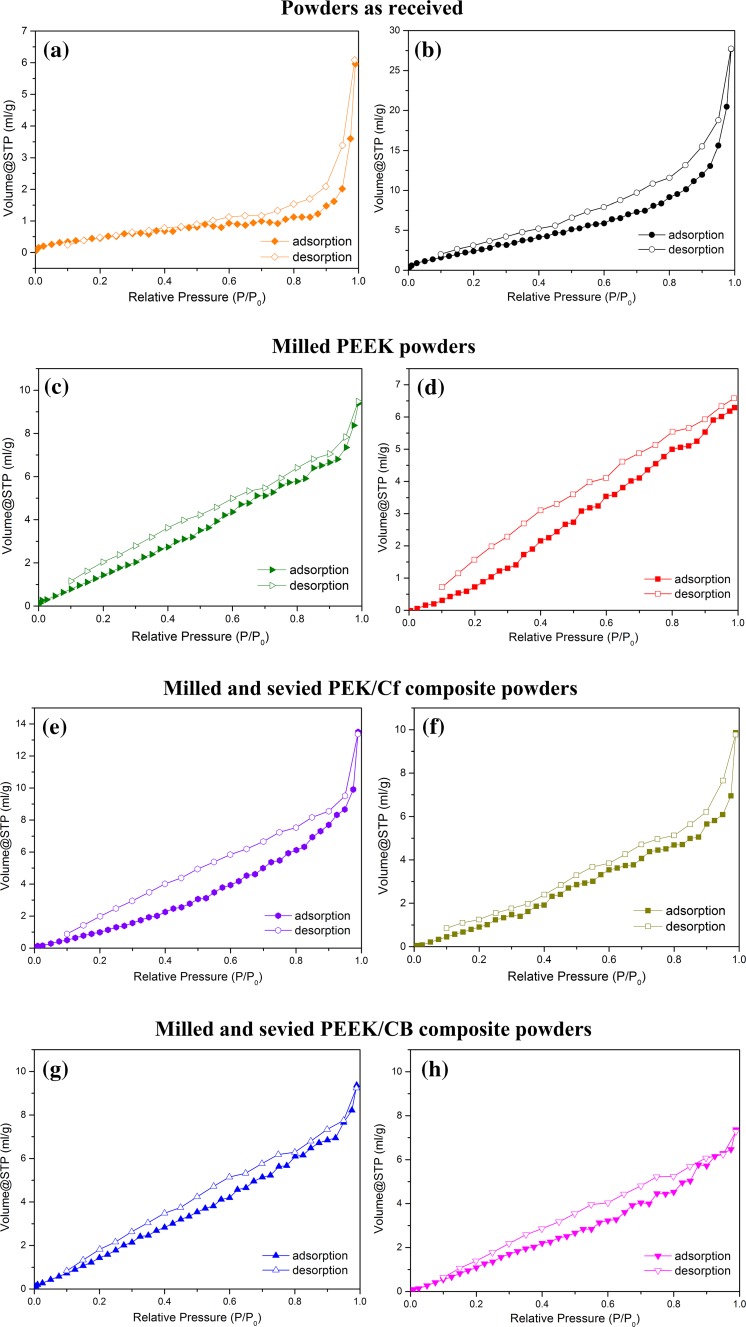
Nitrogen adsorption–desorption isotherm curves of **a** PEK HP3, **b** PEEK 450PF, **c** disc blade milled and **d** rotary knife milled PEEK 450G; **e**, **f** rotary knife milled PEK/Cf composite and **g**, **h** rotary knife milled PEEK/CB (before and after sieving)

The textural properties of PAEK powders and their composite powders were analysed and the results are summarised in Table 3. As shown in Fig. 8, below relative pressure (*P*/*P*_0_) = 0.1, the N_2_ sorption of the powders all exhibit type III isotherm, which is characteristic of non-porous sorbents with low energy of adsorbent-adsorbate interaction. The adsorption–desorption isotherm branches are irreversible, with hysteresis loops between their adsorption and desorption branches. Specific surface area and pore volume values of the powders under investigation are given in Table 3. As the powders are fabricated through various manufacturing methods, their surface areas and densities are different. Hence, PEEK 450PF received little post-processing following polymerisation, which made the particles rougher on the surface and prone to more internal pores and voids. In comparison, PEEK 450G, PEK/Cf and PEEK/CB powders originate from dense melt compounded granules, milled and sieved. These grades have a lower pore volume although the external surface area can remain high, depending on the milling technique employed.

**Table 3:** BET results of PAEK and their composite powders

	Material	Surface area (m^2^ g^−1^)	Pore volume (ml g^−1^)
Powders as received	PEK HP3	1.8	0.01
	PEEK 450PF	11.5	0.04
Milled PEEK powders	Disc blade milled PEEK 450G	9.3	0.02
	Rotary knife milled PEEK 450G	8.7	0.01
Milled and sieved PEK/Cf composite powders	Rotary knife milled PEK/Cf	7.4	0.02
	Sieved PEK/Cf > 63 µm	6.0	0.02
Milled and sieved PEEK/CB composite powders	Rotary knife milled PEEK/CB	9.8	0.01
	Sieved PEEK/CB > 63 µm	9.0	0.01

The optimised commercial powder PEK HP3 has a low surface area, suggesting that the PEK HP3 particles are dense. PEEK 450PF has the highest surface area value, indicating the high amount of voids or pores, as well as a rough particle surface. Disc blade milled PEEK 450PF and rotary knife milled PEEK 450G powders exhibit a 19–24% decrease in specific surface area and a decrease in pore volume, compared to PEEK 450PF, indicating that having dense materials will significantly decrease the surface area and pore volume of the resulting powders. Moreover, it seems that rotary knife milling method is superior to disc blade milling method when fabricating dense powders. Denser powder particles can reduce curling of layers and form smooth layers during LS process; therefore, understanding external and internal surface area of particles is very important.

### Hot-stage microscopy analysis

**Figure 9:**
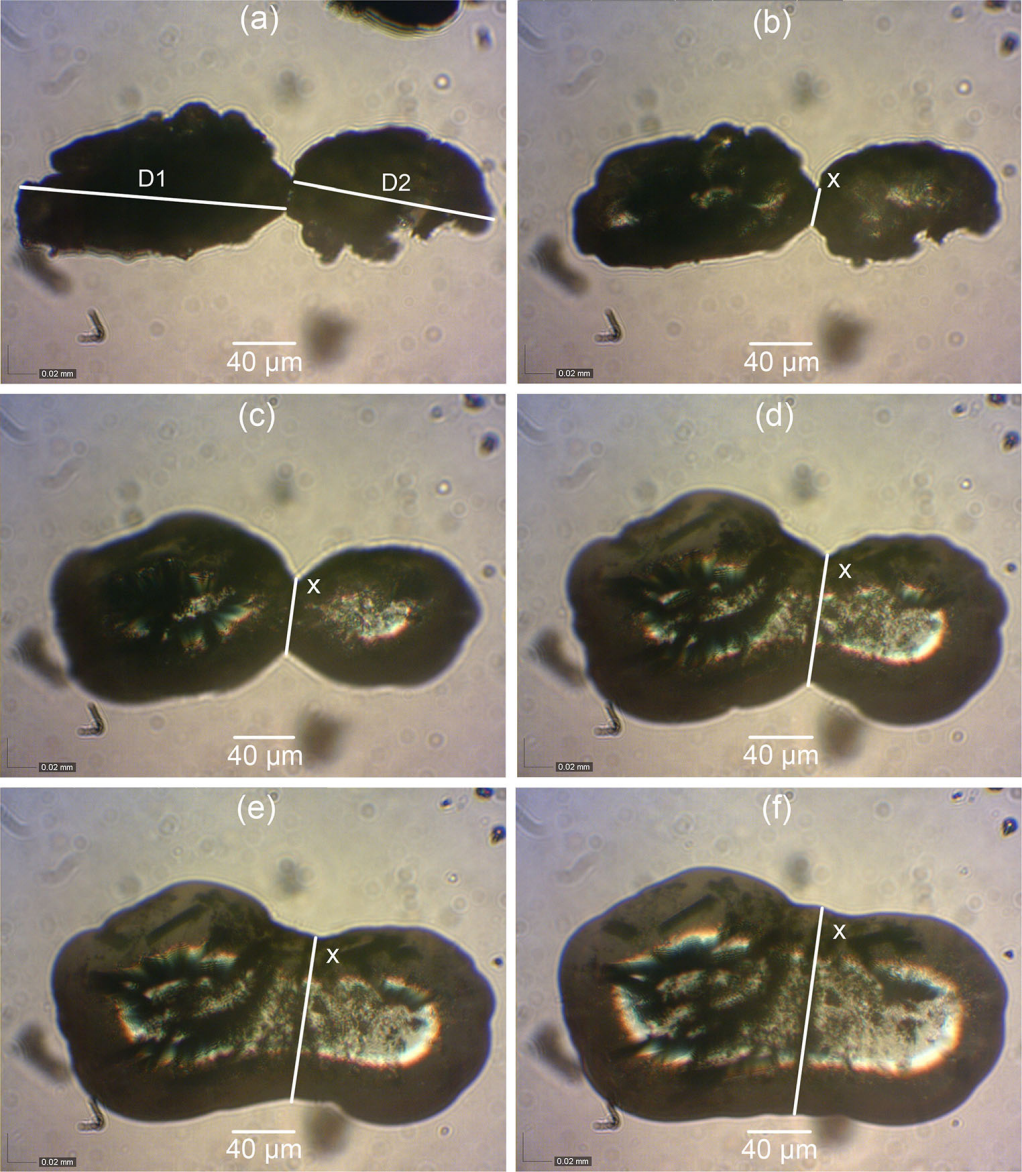
Particle coalescence of sieved PEK/Cf composite: **a** Room temperature, **b**–**f** during coalescence in the temperature ranging from 380 to 400 °C. *D*1 and *D*2 are particle diameters, *D* is (*D*1 + *D*2)/2, and *x* is the neck length

**Figure 10:**
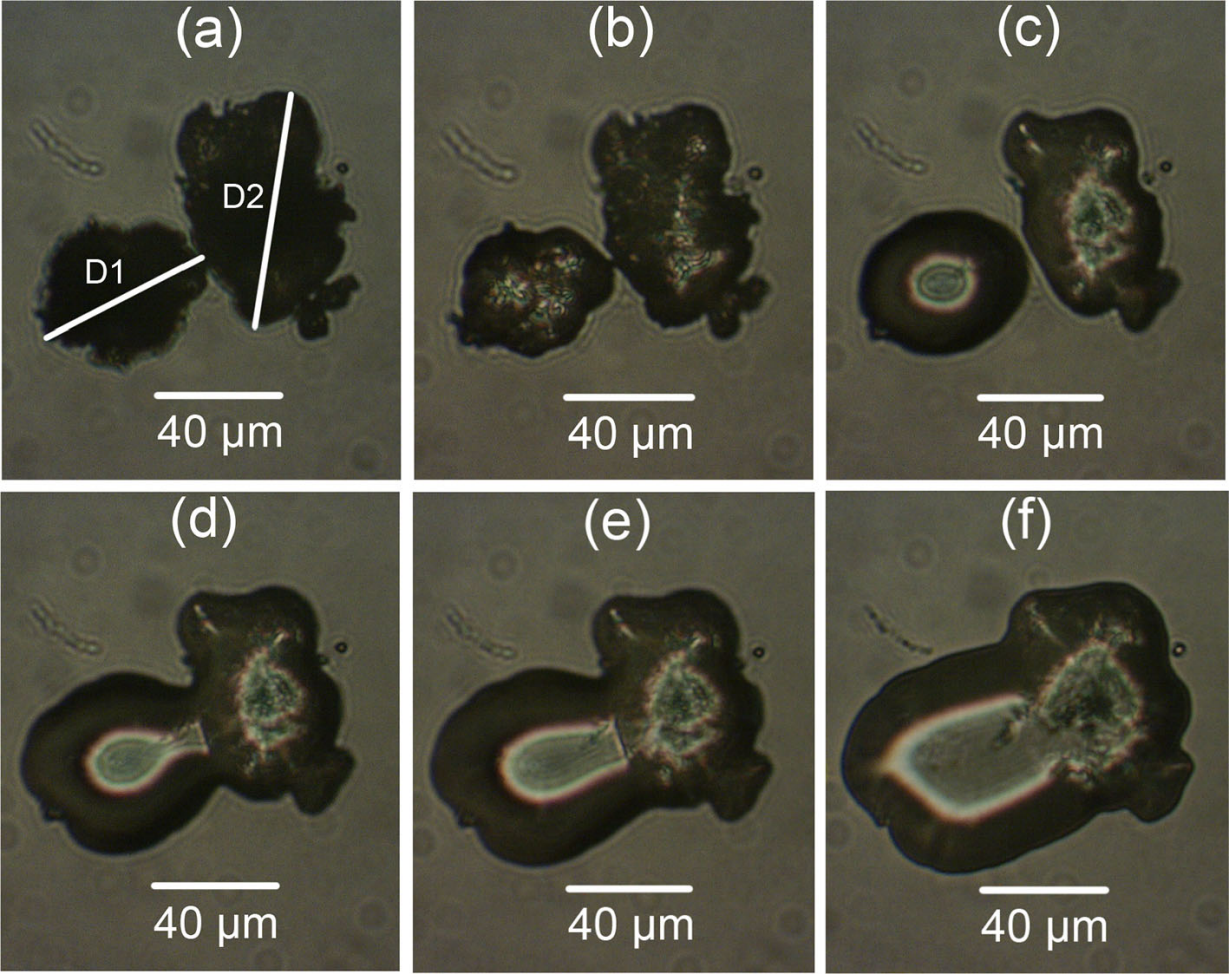
Particle coalescence of PEEK450PF

**Figure 11:**
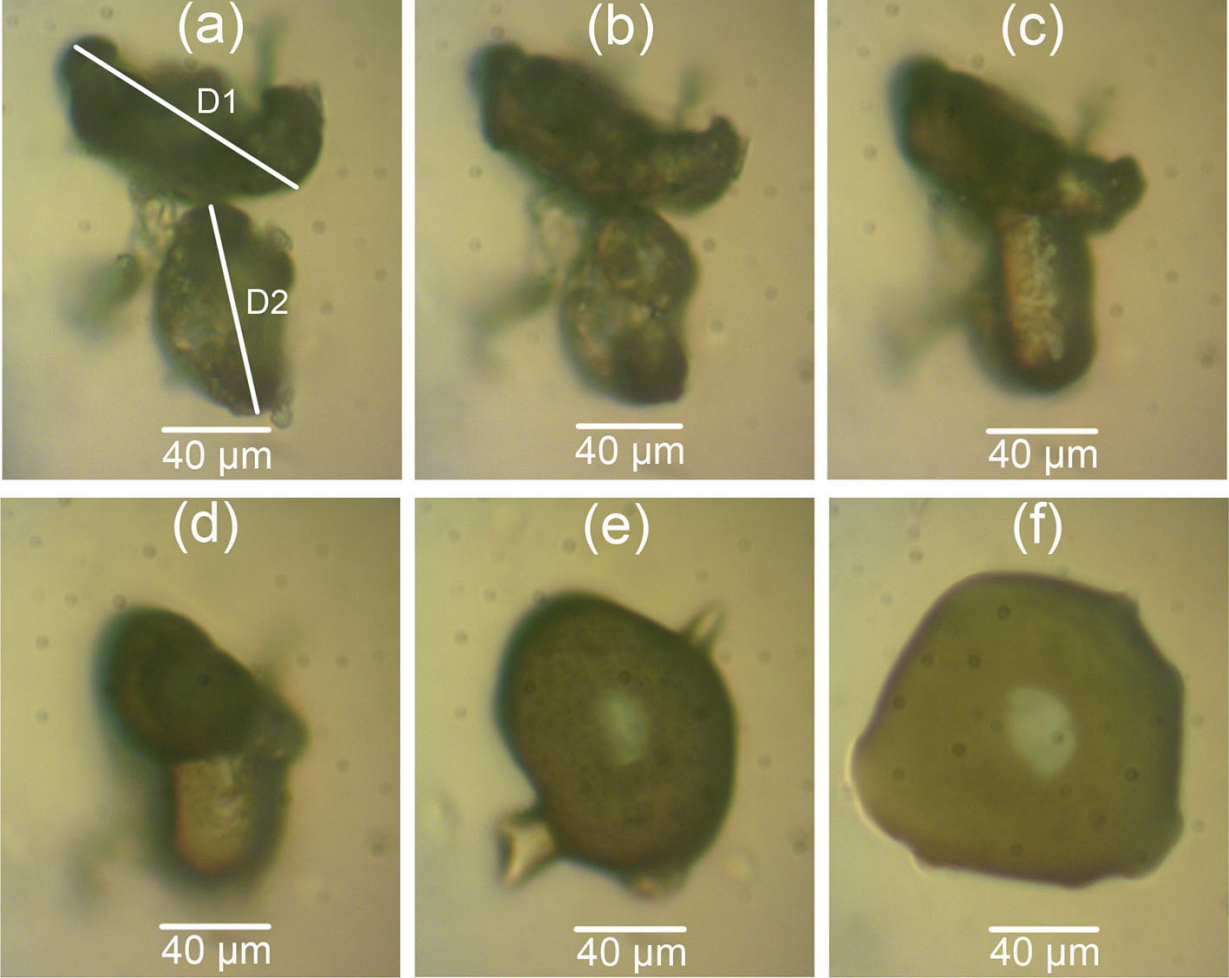
Particle coalescence of sieved PEEK/CB composite

**Figure 12:**
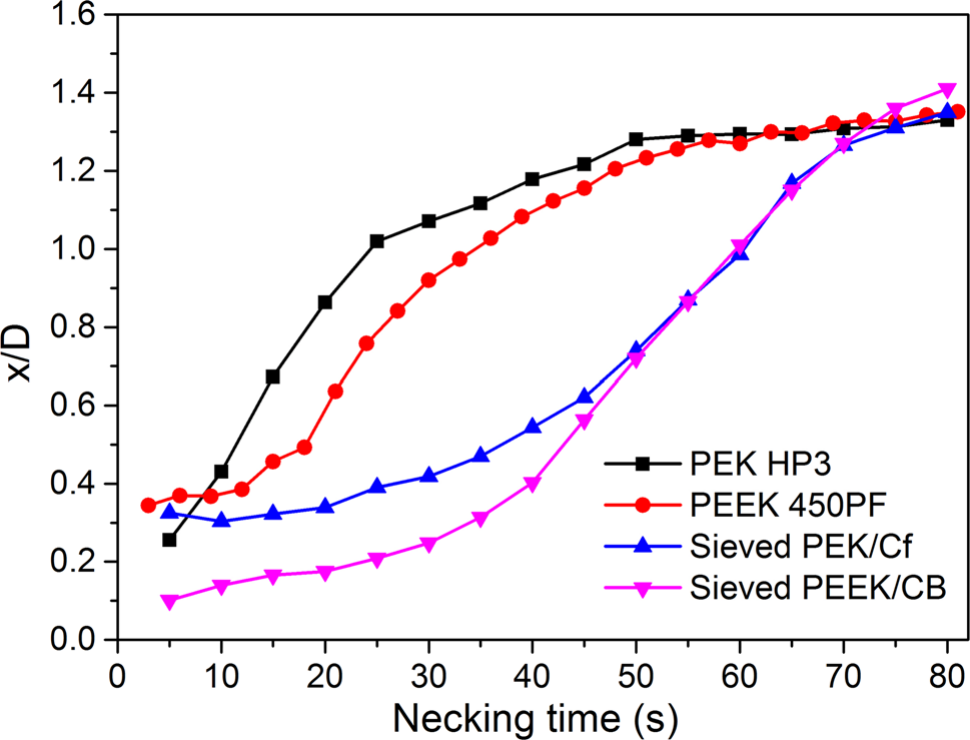
Coalescence results of PEK HP3, PEEK 450PF, sieved PEK/Cf, and sieved PEEK/CB composite particles

The particle diameters and neck length of the sieved PEK/Cf composite are shown in Fig. 9. The hot-stage microscopy for PEEK450PF and sieved PEEK/CB composite are shown in Figs. 10 and 11. The ratio *x*/*D* of these measured powders is plotted against the neck formation time (Fig. 12). For PEK HP3 and PEEK 450PF, the initial part of the neck formation is different, which could be due to the changes in the particle morphology, but they end with a similar neck growth [24]. Compared with PEK HP3 and PEEK 450PF, sieved PEK/Cf composite exhibited a very different curve shape, where the slope of the initial part was low and then the rate of neck growth increases significantly. This change could be due to the thermal conductive property of the carbon fibre present in the PEK particles. Sieved PEEK/CB composite exhibited a similar curve to PEK/Cf. It is evident that the particle morphology and the addition of carbon fibres play an important role in the coalescence process, which can further influence the LS process in operation.

**Figure 13:**
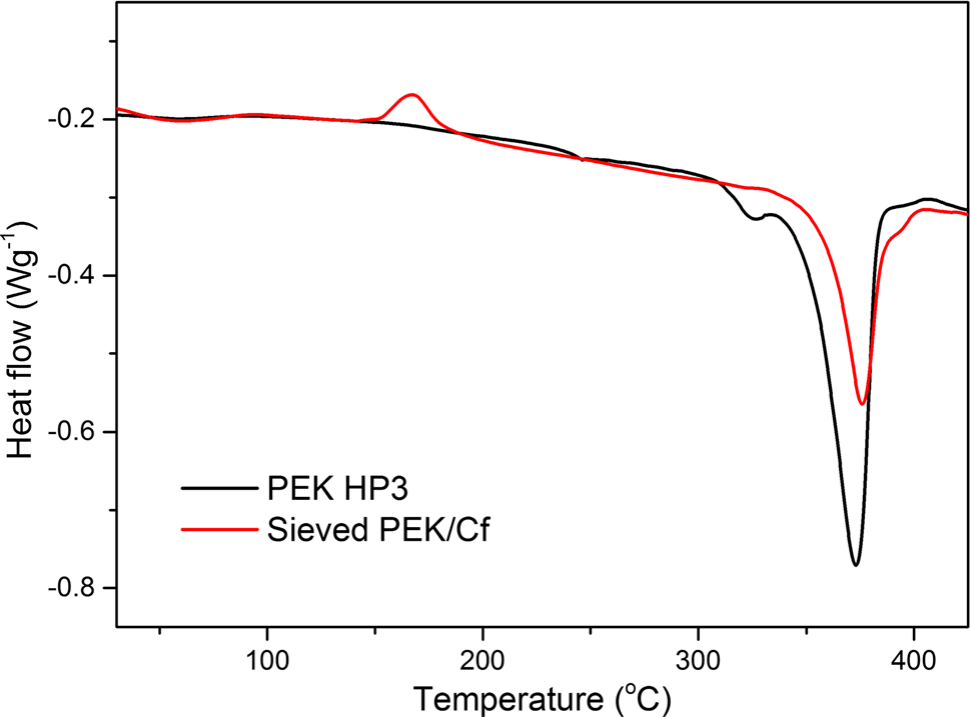
DSC results of PEK HP3 and sieved PEK/Cf

DSC was also carried out for the PEK/Cf and PEK HP3 to evaluate the thermal influence of the carbon fibre on the polymer matrix and coalescence process. Clearly, the melting point of the sieved PEK/Cf powder is higher than that of the PEK HP3 powder. The increase in the melting point of PEK/Cf is in agreement with their coalescence process result, which shows a delay in the start and rate of sintering process. The time delay in the PEK/Cf particle coalescence as seen in Fig. 12 is very important during the laser sintering process, e.g. during multiple laser exposure of the material or simple deposition of the layers of powder (Fig. 13).

## Conclusions

Key properties in the development of polymer and composite polymeric powders for laser sintering have been reported. Particle morphology, circularity, roundness, aspect ratio, angle of repose, and textural properties of the PAEK powders and their composite powders were systematically analysed. It was found that the rotary knife milling method is superior to disc blade milling methods when milling tough polymers such as PAEK and their composites. The sieved PEK/Cf and PEEK/CB composites provide good flowability and processability. The key particle properties of rotary knife milled PEEK 450G, sieved PEK/Cf composite, and sieved PEEK/CB composite appear promising and will represent, through refining, strong material candidates for high-temperature laser sintering.
